# Molecular Epidemiology of SARS-CoV-2 and Clinical Manifestations among Organ Transplant Recipients with COVID-19

**DOI:** 10.3390/v16010025

**Published:** 2023-12-22

**Authors:** Abeer N. Alshukairi, Ahmed A. Al-Qahtani, Dalia A. Obeid, Ashraf Dada, Reem S. Almaghrabi, Maha A. Al-Abdulkareem, Basma M. Alahideb, Madain S. Alsanea, Feda A. Alsuwairi, Fatimah S. Alhamlan

**Affiliations:** 1 Department of Medicine, King Faisal Specialist Hospital and Research Centre, Jeddah 21362, Saudi Arabia; aalshukiri@kfshrc.edu.sa (A.N.A.); a.dada@kfshrc.edu.sa (A.D.); 2 College of Medicine, Alfaisal University, Riyadh 11211, Saudi Arabia; aqahtani@kfshrc.edu.sa; 3 Department of Infection and Immunity, King Faisal Specialist Hospital and Research Centre, Riyadh 11211, Saudi Arabia; moalabdulkareem@kfshrc.edu.sa (M.A.A.-A.); balahideb@kfshrc.edu.sa (B.M.A.); maalsanea@kfshrc.edu.sa (M.S.A.); falsuwairi@kfshrc.edu.sa (F.A.A.); 4 Organ Transplant Center of Excellence, King Faisal Specialist Hospital and Research Centre, Riyadh 11211, Saudi Arabia; obeiddx@gmail.com (D.A.O.); ramaghrabi@kfshrc.edu.sa (R.S.A.); 5 Department of Pathology and Laboratory Medicine, King Faisal Specialist Hospital and Research Centre, Riyadh 11211, Saudi Arabia

**Keywords:** molecular epidemiology, SARS-CoV-2, COVID-19, immunocompromised patients, organ transplant recipients, variant of concern

## Abstract

RNA viruses, including SARS-CoV-2, rely on genetic mutation as a major evolutionary mechanism, leading to the emergence of variants. Organ transplant recipients (OTRs) may be particularly vulnerable to such mutations, making it crucial to monitor the spread and evolution of SARS-CoV-2 in this population. This cohort study investigated the molecular epidemiology of SARS-CoV-2 by comparing the SARS-CoV-2 whole genome, demographic characteristics, clinical conditions, and outcomes of COVID-19 illness among OTRs (*n* = 19) and non-OTRs with (*n* = 38) or without (*n* = 30) comorbid conditions. Most patients without comorbidities were female, whereas most OTRs were male. Age varied significantly among the three groups: patients with comorbidities were the oldest, and patients without comorbidities were the youngest. Whole-genome sequencing revealed that OTRs with mild disease had higher numbers of unusual mutations than patients in the other two groups. Additionally, OTRs who died had similar spike monoclonal antibody resistance mutations and 3CLpro mutations, which may confer resistance to nirmatrelvir, ensitrelvir, and GC37 therapy. The presence of those unusual mutations may impact the severity of COVID-19 illness in OTRs by affecting the virus’s ability to evade the immune system or respond to treatment. The higher mutation rate in OTRs may also increase the risk of the emergence of new virus variants. These findings highlight the importance of monitoring the genetic makeup of SARS-CoV-2 in all immunocompromised populations and patients with comorbidity.

## 1. Introduction

When the World Health Organization announced that the coronavirus disease 2019 (COVID-19) outbreak was a pandemic, Saudi Arabia embraced their COVID-19 response recommendations, including vaccinations. By 10 May 2023, a total of 69,635,799 doses of vaccine had been given in Saudi Arabia alongside a reported total of 841,469 confirmed cases of COVID-19, with 9646 fatalities [1]. During the initial phase of the COVID-19 outbreak, the variety of symptoms and imaging findings, as well as the severity of the disease at presentation, complicated the diagnosis of the disease. Since then, a plethora of studies have been conducted worldwide to characterize the virus and to study its risk factors and prognosis [2]. For example, older adults and individuals with comorbid conditions, in particular those with cardiovascular disease, diabetes, and obesity, were found to be at a higher risk for infection and adverse outcomes [3]. Although hundreds of studies have reported research findings associated with the virus variant waves, few have assessed COVID-19 outcomes among organ transplant recipients (OTRs) [4,5,6,7,8].

It is uncertain whether immunosuppression constitutes a risk factor for poor COVID-19 outcomes. Findings of previous studies involving OTRs have indicated a poorer prognosis and a higher mortality rate than the hospitalized general population but have not taken into account the different comorbid conditions among OTRs [4]. To better understand COVID-19 outcomes among OTRs, recent studies have used logistic regression analyses for large populations of hospitalized patients and matched cohorts. After adjusting for demographic characteristics and a number of comorbid conditions, one study reported higher morbidity for OTRs, manifested by the need for mechanical ventilation, in the total study population and a trend toward an increased combined end point of death or mechanical ventilation [4]. Due to chronic immunosuppressive therapy and other medical complications, OTRs may be at high risk for COVID-19 infection; mortality rates for OTRs are reportedly between approximately 13% and 30%.

Several studies using the longitudinal sampling of small numbers of patients have reported the estimated duration of SARS-CoV-2 viral shedding in immunocompromised patients. These studies consistently found that immunocompromised patients with COVID-19 had extended periods of viral shedding that could last several months. Therefore, immunocompromised patients must be de-isolated with caution [5,6,7]. Immunocompromised patients are more likely to be long-term virus carriers, which increases the probability of subsequent coinfections and recombination events that lead to the generation of variants [8,9,10]. Additionally, the clinical ramifications of COVID-19 infection may vary based on the type of transplanted organ and recipient comorbidities, which would influence the decision to continue transplantation during the pandemic. Hence, the selection of both donors and recipients for transplantation must be tailored during a pandemic. Moreover, although great strides have been made in COVID-19 treatment strategies and vaccinations, their impact may be diminished among OTRs due to their immunosuppression [11,12].

The King Faisal Specialist Hospital and Research Centre (KFSHRC), a tertiary hospital in Saudi Arabia that provides specialized care for OTRs in addition to other medical specialties, has been conducting epidemiologic and genomic surveillance to monitor the circulating virus variants of concern (VOCs). This study leveraged these surveillance data and conducted SARS-CoV-2 whole-genome sequencing to monitor the spread and evolution of SARS-CoV-2. We found that, compared with patients without transplants with or without comorbidities, the OTRs in this study had higher numbers of unusual mutations, which may increase the risk for the emergence of new virus variants. These unusual mutations may also affect the virus’s ability to evade the immune system or to respond to treatment, which could impact the severity of COVID-19 illness. Overall, our findings provide valuable insights into the geographical spread of the virus, the emergence of novel variants, and the efficacy of various interventions in immunocompromised populations.

## 2. Materials and Methods

### 2.1. Collection of Samples and Clinical and Demographic Data

In total, 92 PCR-confirmed SARS-CoV-2–positive patient nasopharyngeal samples were collected from the Pathology and Laboratory Medicine Department at KFSHRC, Jeddah, between 1 April 2021 and 31 March 2022. Demographic characteristics, vaccination history, comorbidities, symptoms, laboratory reports, and intensive care unit (ICU) admissions obtained from electronic health records were evaluated as variables. After passing sample integrity quality control metrics, all PCR-confirmed SARS-CoV-2–positive samples that met inclusion criteria were sequenced for the SARS-CoV-2 whole-genome and were processed and analyzed by next-generation sequencing. Only data from samples that passed the quality control criteria were included in this study. All sequences were submitted to GISAID.

### 2.2. Ethics Approval

The Research Advisory Council (Clinical Research Committee) at KFSHRC reviewed and approved this study (RAC #220 0009).

### 2.3. Sample Preparation

RNA was extracted from 200 μL of the nasopharyngeal sample using the MagMAXTM Viral/Pathogen II Nucleic Acid Isolation Kit (Thermo Fisher Scientific, Waltham, MA, USA). The RNA integrity was evaluated using a Nanodrop system, with a 260/280 ratio of approximately 2.00 deemed acceptable. The nucleocapsid, open reading frame (ORF)1ab, and spike genes were targeted by real-time PCR using TaqPathTM COVID-19 CE-IVD RT-PCR Kits (Thermo Fisher Scientific, Waltham, MA, USA) according to the manufacturer’s instructions. Real-time PCR was performed utilizing a 7500 Fast Real-Time PCR system and associated software (Applied Biosystems, Foster, CA, USA). All SARS-CoV-2–positive samples were converted to cDNA using SuperScriptTM IV VILOTM Master Mix (Thermo Fisher Scientific, Waltham, MA, USA).

### 2.4. Sequencing and Bioinformatics Analysis

Using an Ion GeneStudio S5 System (Thermo Fisher Scientific, USA), we performed next-generation sequencing. By following the manufacturer’s instructions, we amplified cDNAs with the Ion AmpliSeqTM SARS-CoV-2 Insight Research Assay. Using the Ion Xpress Barcode Adaptors 1–16 kit, the amplified products were ligated with unique barcode adaptors and purified with Agencourt AMPure XP Reagent (1.5; Beckman Coulter, Brea, CA, USA). The constructed libraries were normalized to 33 pM with nuclease-free water, and up to 16 libraries were pooled equally. The pooled libraries were used as templates for emulsion PCR and enrichment of template-positive particles using the Ion Chef automated system with the Ion 510 & Ion 520 & Ion 530 Kit-Chef kit per the manufacturer’s instructions. Enriched template-positive ion sphere particles were loaded onto an Ion 530 chip, and sequencing was conducted using an Ion GeneStudio Ion S5 sequencer. The obtained data were primarily processed (base calling and quality, alignment, assembly, and variant calling) with Torrent SuiteTM software using Torrent Server version 5.12. The contigs were assembled from scratch using the assembly Trinity plugin (v1.2.1), and consensus sequences of each sample were generated using the IRMA plugin (v1.2.1). The variant call files were analyzed with the COVID-19 SnpEff plugin to identify and annotate those in public and private databases.

### 2.5. Phylogenetic Analysis

The FASTA file names we utilized were annotated with patient information (sample identification, variant, month of infection, vaccinated or unvaccinated, healthcare worker or not, and ICU admission or regular hospital admission). The sequences were aligned against the reference (NC 045512.2) using MAFFT (v 7.490) and the 6merpair method to generate a consensus alignment file [13]. We used the maximum likelihood method with the IQ-TREE tool (v2.2.0-beta COVID-edition) for phylogenetic analyses and used the ModelFinder tool (within IQ-TREE) to determine that the TIM2 + F + I nucleotide substitution model was the optimal model for our samples [14,15]. We utilized FigTree software (v1.4.4) (http://tree.bio.ed.ac.uk/software/figtree/, (accessed on 7 February 2023)) to visualize the phylogenetic trees. All generated sequences were uploaded to the GISAID database.

### 2.6. Statistical Analysis

All acquired data were stored and analyzed using SAS 9.4 and Prism 3.0. (GraphPad). Clinical factors were evaluated using inferential and descriptive statistics, continuous variables were evaluated using an analysis of variance or *t*-tests, and categorical variables were evaluated using the chi-square test. Univariate and multivariate logistic regression models were performed to determine the odds ratios for ICU admission by patient clinical and demographic factors. A global *p*-value for each variable is reported. All reported *p*-values are two-tailed, and a value of 0.05 was deemed statistically significant.

## 3. Results

### 3.1. Patient Demographic and Clinical Characteristics

Of 92 patient samples collected from 1 April 2021 to 31 March 2022, 5 had missing metadata and were not included in the further analyses. Thus, all analyses were conducted using data from 87 patients. The overall mean patient age was 44.2 years (SD = 20.4 years), with a minimum age of 5 months and a maximum age of 90 years. Most patients were female (56.3%; 43.7% male). Only 9.2% of patients reported a history of or current tobacco smoking. By the study’s end (31 March 2022), 10 patients died, 61 patients recovered, 1 patient was still hospitalized, and 15 were released from the hospital. Regarding COVID-19 disease severity, 79.5% of patients had mild disease and 20.5% had severe illness. Most patients were treated as outpatients (77.9%), with only 22.1% treated in the hospital. In total, 18.4% of patients were admitted to the ICU.

The cohort comprised 38 (43.7%) patients with comorbidities, 30 (34.5%) with no comorbid condition, and 19 (21.8%) with organ transplants. Most OTRs received a kidney (78.9%), followed by a liver (15.8%). One receipt of a bone marrow transplant developed pancytopenia. Of the reported patient comorbidities, 38.4% were immunocompromised conditions, 27.6% were diabetes, and 36.8% were hypertension.

The variants detected in the overall cohort were Omicron (69.6%), Delta (18.5%), non-VOCs (4.4%), Kappa (3.3%), Alpha (2.2%), and Beta (2.2%). We separated the cases by Delta and Omicron waves: cases detected before 1 January 2022 were considered part of the Delta wave (28.3% of cases), and cases detected after that date were considered part of the Omicron wave (71.7% of cases).

Regarding vaccination data, 87.5% of patients were vaccinated before being infected with COVID-19, and 12.5% were not vaccinated. The most common vaccine received in this cohort was from Pfizer-BioNTec (58.34%), followed by AstraZeneca (30.6%). Most of the vaccinated patients received two doses (57.5%), with fewer receiving a booster (25.0%) or only one dose (17.5%).

### 3.2. Comparisons of Clinical and Demographic Characteristics among OTRs and Patients without Organ Transplant with or without Comorbid Conditions

**Figure 1:**
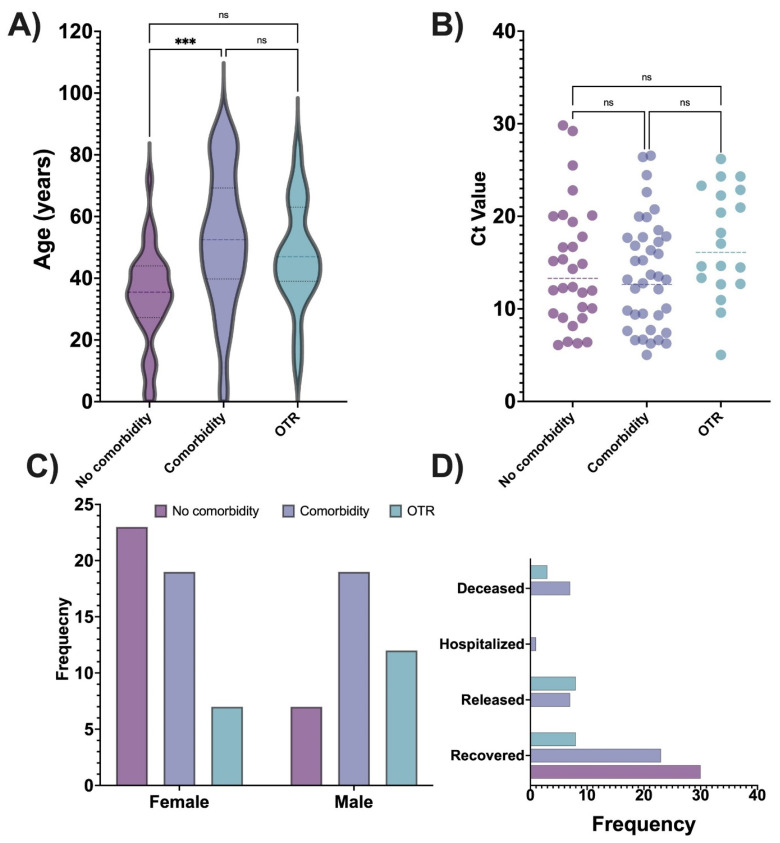
Key clinical and demographic characteristics in organ transplant recipients (OTRs) compared with patients without organ transplant with or without comorbid conditions. (**A**) Box plot of age distribution by the patient group. Dashed horizontal lines represent the mean; upper and lower dotted lines represent 95% CI. The oldest mean age was found in the group with comorbid conditions, followed by OTRs, and then the group without comorbidities. (**B**) Box plot of the cycle threshold (Ct) for the PCR test by patient group. Circles represent individual patient data; horizontal dash lines represent the mean. The lowest mean Ct value (suggesting the highest viral load) was found in patients with comorbid conditions, followed by patients without comorbidity, and then OTRs. (**C**) Bar graph of patient groups by sex. Organ transplant was most common among males. (**D**) Bar graph of the distribution of patient groups by outcome. Most released patients were in the group without comorbid conditions. Most deceased patients had a comorbid condition, followed by an organ transplant. *** Indicates statistical significance at *p* < 0.0001; ns, not significant.

To better understand COVID-19 disease in OTRs, we compared the demographic and clinical characteristics of OTRs to patients without organ transplants with or without comorbid conditions. A summary of this analysis is given in Table 1. A significant association was found in the patient group for sex—OTRs were mostly male, whereas patients without comorbidities were mostly female—and age, with the oldest patients having comorbidities and the youngest patients having no comorbidities. Patient outcome was also significantly different: all patients without comorbid conditions recovered, whereas most of the deceased patients had comorbidity. Among 19 OTRs, most recovered, a few were released from the hospital, and 3 died. There were significant group differences by disease severity, with severe disease detected only among patients with comorbidity or OTRs. Hospital admission was significantly associated with the patient group: most hospitalized patients had a comorbid condition. No patient without comorbidity required an extended length of hospital stay, whereas patients with comorbid conditions had the longest hospital stay, followed by OTRs. The ICU admission differed by patient group, with most admitted patients having comorbid conditions, followed by OTRs. Diabetes, hypertension, or immunocompromised were most common among OTRs. The threshold cycle (Ct) value for PCR (an indicator of the amount of viral genetic material detected in a sample) was significantly different by patient group, with the lowest Ct values (suggesting higher viral loads) detected in patients with comorbid conditions and patients without comorbid conditions. By contrast, no significant difference was detected between groups by either vaccination type or dose. Figure 1 box plots and bar charts show the distribution of the key variables by the patient group.

### 3.3. Demographic and Clinical Characteristics of the Entire Cohort by Variant Wave

This study also assessed whether specific demographic or clinical characteristics of patients with COVID-19 differed by the Delta or Omicron wave. A summary of the analysis is given in Table 2. No significant difference between the two variant waves was detected for sex, age, and COVID-19 outcome. OTRs were the most common patient group during the Delta wave, whereas patients without organ transplants with or without comorbid conditions were the most common group during the Omicron wave. Diabetes was significantly different between the two waves, with a higher percentage of patients having diabetes as a comorbidity during the Delta wave. Another difference between the two variant waves was detected for vaccination status, with most patients in the Omicron wave having been previously vaccinated. A significant association was also detected by vaccine dose: none of the patients in the Delta wave received a booster.

### 3.4. Phylogenetic and Outbreak Analyses for Omicron and Delta Waves in the Entire Cohort

To understand the COVID-19 outbreaks in KFSHRC, we conducted a phylogenetic analysis using IQ-TREE software and a model finder. We built two phylogenetic trees, one for the Delta wave and the other for the Omicron wave. We included data only from patients with good-quality samples and high coverage (i.e., a high number of sequencing reads that were uniquely mapped to a specific locus in a known portion of the reference genome).

**Figure 2:**
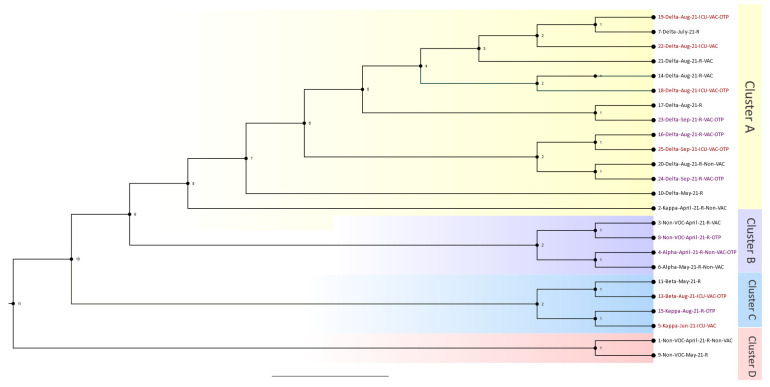
Phylogenetic analysis of all Delta variant outbreaks from 1 April 2021 to 31 January 2022. The information shown on the right of the figure reflects patient data in the following order: sample identification number, variant type, month and year of infection, regular hospital (R) or intensive care unit (ICU) admission, vaccinated (VAC) or unvaccinated (UV), and organ transplant patient (OTP). There were 24 patients, and the estimated tree was constructed using the TIM + F + I + I + R2 model. The estimated branch length is shown for each node.

The Delta wave phylogenetics analysis consisted of 24 out of the 26 patients who passed bioinformatic quality checks. Samples were collected from 1 April 2021 to 31 January 2022. The resulting phylogenetic tree with four distinguished main clusters is shown in Figure 2. Cluster A consisted of six patients from the organ transplant group, four patients from the ICU-admitted group, and one patient from the regular hospital-admitted group (i.e., not admitted to the ICU). Case 19 was a patient admitted to the ICU who was close to a regular hospital case that was a patient who was connected to another patient admitted to the ICU. Case 18 was another patient admitted to the ICU who was an OTR and close to a regular hospital case. The other sub-branch in Cluster A comprised four OTRs and one other patient who was admitted to the ICU. Overall, Cluster A was considered a hotspot, with many cases from the OTR and ICU groups connected. Cluster B contained two OTRs who were very close to each other and regular hospital cases. In cluster C, two cases were OTRs, and two cases were admitted to the ICU. These cases were very close to one another, starting with a case in May (case 11) and progressing to cases 13, 15, and 5. The last cluster, D, comprised two regular hospital cases, and none were OTRs.

**Figure 3:**
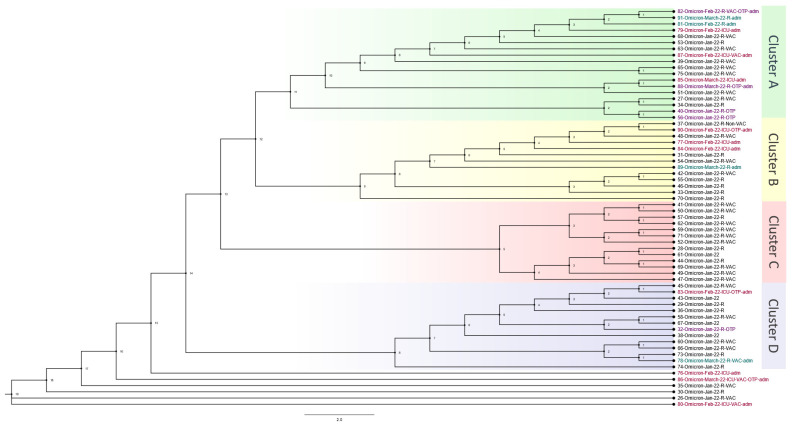
Phylogenetic analysis of all Omicron outbreaks from 1 January 2022 to 31 March 2022. The information shown to the right of the figure reflects patient data in the following order: sample identification number, variant type, month and year of infection, regular hospital (R) or intensive care unit (ICU) admission, vaccinated (VAC) or unvaccinated (UV), and organ transplant patient (OTP). There were 64 patients, and the estimated tree was constructed using the TN + F + I + I + R2 model. The estimated branch length is shown for each node.

The Omicron wave phylogenetics analysis consisted of 64 out of 66 patients who passed bioinformatic quality checks. Samples were collected from 1 January 2022 to 31 March 2022. The phylogenetic tree with four distinguished main clusters and a few unclassified close cases is shown in Figure 3. Cluster A included four OTRs, three other cases admitted to the ICU, and two other cases admitted to the hospital. Case 82, at the top of Cluster A, was an OTR who was close to two hospital-admitted patients and one ICU-admitted patient. Case 87 was an ICU case in the same cluster, and this case was close to a regular hospital case. Another connection was found between an ICU case (85) and an OTR case (88). Furthermore, the same cluster showed two OTR cases (40 and 56) that were very close. Cluster B had one OTR case, three ICU cases, and one hospital-admitted case. These cases occurred close together but with regular hospital cases between them. In Cluster C, none of the cases had been admitted to the hospital or ICU, and none was an OTR. In Cluster D, two OTR cases were detected. One of them was admitted to the regular hospital and one was admitted to the ICU. The final cases in the phylogenetic analysis consisted of three ICU cases, two of which were OTRs. These cases were further apart from the rest of the cases, indicating that they may have been from outside the hospital.

### 3.5. Comparison of SARS-CoV-2 Mutation Profiles in OTRs and Patients without Organ Transplant with or without Comorbid Conditions

**Figure 4:**
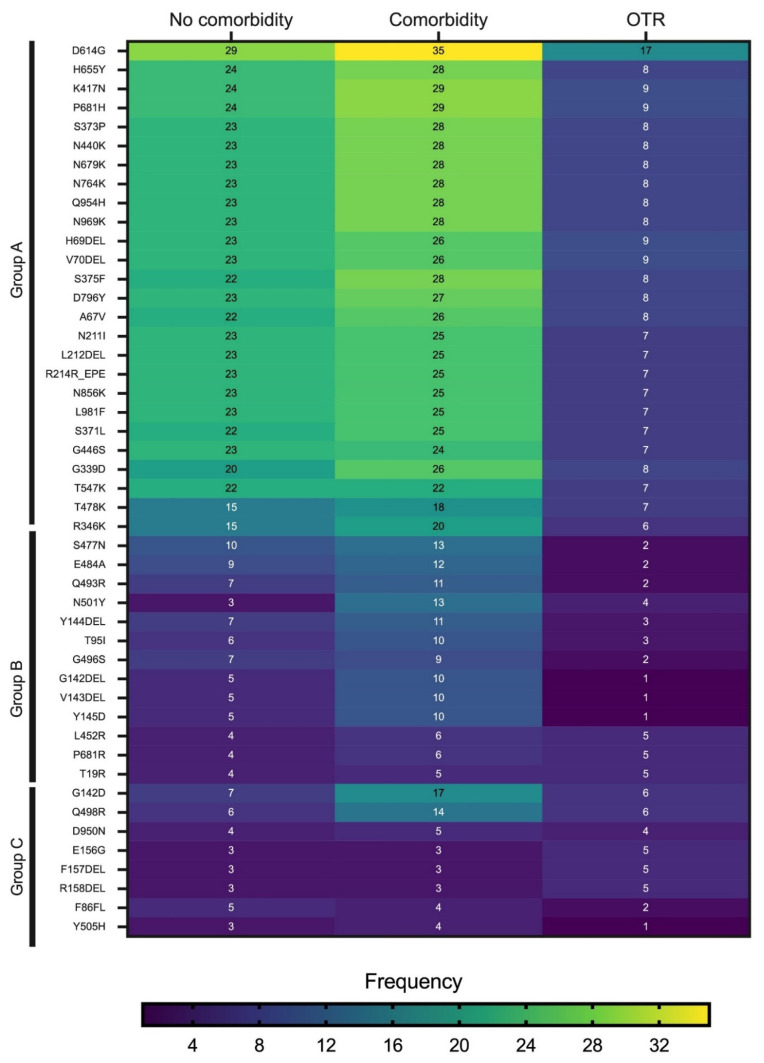
Heatmap of the mutations most frequently detected in the cohort by the patient clinical group. The mutations were separated into three Groups, A, B, and C, based on their frequency of detection, with Group A composed of mutations detected most frequently. The most frequent mutations are shown in yellow and the least frequent in purple. OTR represents organ transplant recipient.

The heatmap in Figure 4 shows the mutations detected with a frequency ≥8 in samples derived from all clinical groups combined. The mutations were separated into three major groups (A, B, and C) based on their overall frequency of detection. Group A comprised the most frequently observed mutations and included D614G, H655Y, K417N, P681H, S373P, N440K, N679K, N764K, and Q954H. The mutations in Group A had the highest frequency of detection in patients with comorbid conditions. The mutations in Group B were least frequently detected in OTRs, followed by patients with comorbidities. Group C comprised the least frequently observed mutations. Three mutations in Group C—E156G, F157DEL, and R158DEL—were observed more frequently in OTRs than in the other two clinical groups. Across all three clinical groups, the mutation detected most frequently was D614G.

**Figure 5:**
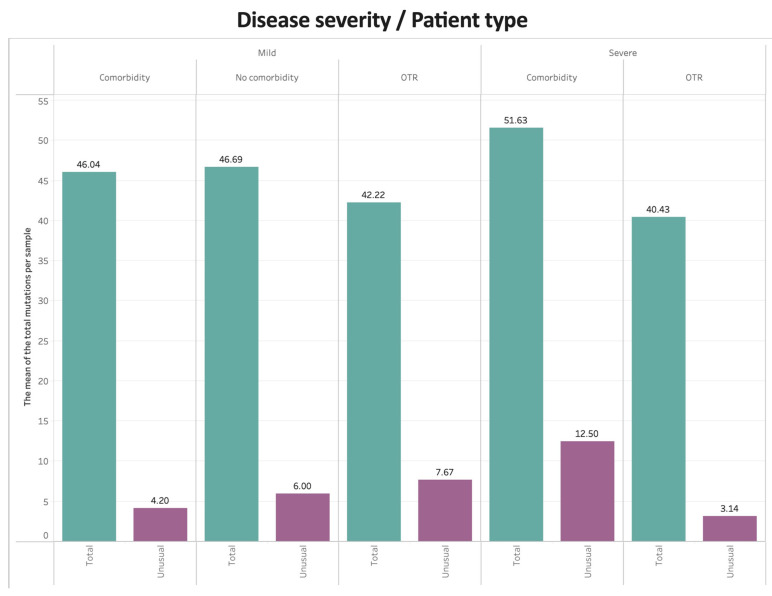
Bar graph of the mean number of total mutations (green bars) and unusual mutations (purple bars) per sample by COVID-19 disease severity and clinical group. For mild disease, patients with or without comorbidities had higher numbers of total mutations compared with OTRs, whereas OTRs had more unusual mutations compared with patients with or without comorbidities. For severe disease, patients with comorbid conditions had the highest numbers of both total and unusual mutations. Mutations were sorted using the Stanford Coronavirus Antivirus and Resistance Database.

The type of mutations and the mean number of mutations detected were sorted using the Stanford Coronavirus Antivirus and Resistance Database. For patients with comorbidity, the average detected mutations per sample was 47.4, while the unusual detected mutations averaged 6.2 per sample. For the OTR group, the average detected mutations per sample was 41.4, while the unusual detected mutations averaged 5.7 per sample. For patients with no comorbidity, the average detected mutations per sample was 46.7, while the unusual detected mutations averaged 6.0 per sample. Figure 5 shows the mean number of total mutations and unusual mutations per sample by COVID-19 disease severity and clinical group. For mild disease, patients with or without comorbidities had higher numbers of total mutations compared with OTRs, whereas OTRs had more unusual mutations compared with patients with or without comorbidities. For severe disease, patients with comorbid conditions had the highest numbers of both total and unusual mutations.

**Figure 6:**
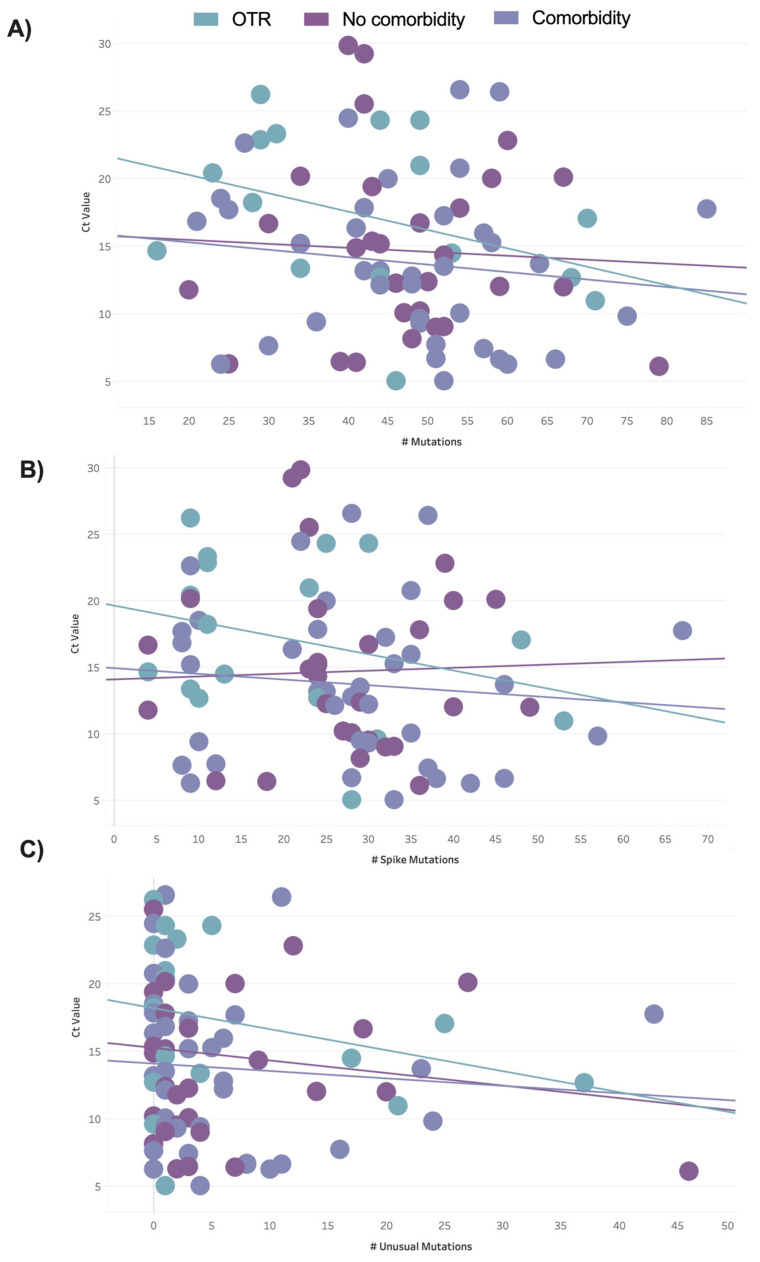
Scatter plot of the relationships between the average cycle threshold (Ct) per sample and the detected mutations by patient clinical group. (**A**) Ct values vs. all detected mutations (R^2^ values: OTR = 0.14, with comorbidity = 0.017, and without comorbidity = 0.003, *p* > 0.05). (**B**) Ct values vs. all detected spike mutations (R^2^ values: OTR = 0.08, with comorbidity = 0.01, and without comorbidity = 0.0014, *p* > 0.05). (**C**) Ct values vs. unusual mutations detected (R^2^ values: OTR = 0.081, with comorbidity = 0.006, and without comorbidity = 0.021, *p* > 0.05).

To evaluate the relationship between the Ct value and the mutations per sample, we used linear regression analyses and displayed the results as scatter plots (Figure 6). The plot of the total number of mutations detected per sample against the Ct value by patient clinical group (Figure 6A) showed a more positive albeit not statistically significant relationship for OTRs compared with patients with or without comorbid conditions. For OTRs, lower Ct values (higher viral load) were observed for cases with higher numbers of mutations. A plot of the total spike mutations detected per sample against Ct values showed similar results (Figure 6B). A plot of the Ct values against the number of unusual mutations (Figure 6C) showed that most patients had zero unusual mutations. The highest number of unusual mutations was observed in one patient without comorbid conditions followed by one patient with comorbid conditions.

A summary of the SARS-CoV-2 variants detected in OTRs that have been associated with antiviral treatment resistance is given in Table 3. We used the Stanford Coronavirus Antiviral and Resistance Database to assess the susceptibility of SARS-CoV-2 mutations to this resistance [16]. Of the 19 samples from OTRs, 2 samples were removed for poor quality, thus, 17 samples with good quality were used for this analysis. For spike mutations associated with monoclonal antibody resistance, every variant had a different set of mutations. For samples with Alpha and Beta variants, the N501Y mutation was very common. For Delta samples, L452 was the most common mutation. For Omicron samples, the following sets of mutations were very common: R346K, S371L, K417N, N440K, G446S, N856K, and N969K. Table 3 also shows 3C-like proteinase (3CLpro) mutations, which refer to mutations selected in a VSV-based system that confer resistance to nirmatrelvir, ensitrelvir, and GC37. The most common of these mutations detected in OTRs was P132H, which was found primarily in Omicron samples. Another group of mutations was found in the RNA-dependent RNA polymerase (RdRP) region, with the most common mutation being P323L, which was found in most samples. The total numbers of mutations in the OTR group were the lowest in non-VOC samples (*n* = 19 mutations) and highest in Omicron samples (mea*n* = 53.3 mutations). The highest number of unusual mutations was detected in Alpha samples (*n* = 37 mutations), followed by one Omicron case (*n* = 25 mutations). Overall, the heaviest load of mutations in the OTR group was detected in Omicron samples, with a mean of 32.8 mutations per sample. Only two samples had a high number of unusual spike mutations, and both were Omicron samples. Deceased patients of the OTR group had no unusual mutations and had similar profiles of mutations for spike monoclonal antibody resistance mutations, RdRp mutations, and 3CLpro mutations. One of the three OTR deceased patients had the Delta variant, and the other two had the Omicron variant. The patient infected with the Delta variant was 49 years old and transplanted a kidney post-hepatitis C infection cure. This patient was treated with methylpresolone, tocilizumab, and other antimicrobial drugs; he was hospitalized for more than 33 days, and the cause of death was septic shock and COVID-19 pneumonia. While the Omicron patients were 63 and 70 years old, they had transplanted kidneys and liver and were hospitalized for 47 and 94 days, respectively. Both of these patients had severe comorbidities; the first one was treated with methylpresolone, and the other one was treated with methylpresolone, tocilizumab, and remdesivir. We also conducted an analysis of all deceased patients, including OTRs and patients with comorbid conditions. We found that most of the Omicron samples obtained from patients who later died showed similar profiles for spike monoclonal antibody resistance mutations, 3CLpro mutations, and RdRp mutations (Table 4).

## 4. Discussion

Molecular epidemiology studies of SARS-CoV-2 have been critical in understanding the transmission, evolution, and pathogenesis of the virus. These studies have shed light on the emergence and spread of multiple lineages and variants of the virus, including the highly transmissible Delta variant. A recent review article emphasized the importance of studying the characteristics, prevalence, and patterns of SARS-CoV-2 infections to monitor and control the pandemic [17]. In addition, the continuous emergence of new variants makes it crucial to implement genomic epidemiology and phylogenetic methods for molecular monitoring and surveillance of the virus. The profile of the pandemic may change rapidly when new variants emerge and spread, impacting epidemiology and public health. Additionally, molecular epidemiology studies have provided insights into the origin and evolution of the virus as well as its transmission dynamics. Such studies have helped to identify potential sources of infection and to understand the role of asymptomatic carriers in the spread of the virus.

The use of whole-genome sequencing and other molecular techniques has enabled researchers to detect mutations in the virus and monitor their prevalence over time, which has important implications for vaccine development and public health interventions. In the present investigation, whole-genome sequencing was conducted to compare the viral mutations in OTRs with those in patients without organ transplants with or without comorbidities. The results showed that OTRs had a higher number of unusual mutations with mild disease than the other two clinical groups. This finding suggests that the genetic makeup of the virus may be more diverse in OTRs. This increased virus diversity may be due to immunosuppression and prolonged exposure to the virus. These unusual mutations could have implications for the severity of COVID-19 in OTRs, as they may affect the virus’s ability to evade the immune system or respond to treatment. Additionally, the higher mutation rate in OTRs could potentially increase the risk of the emergence of new variants of the virus.

Few research articles have been published that report molecular epidemiology in OTRs. A recent article [18] shared their analysis of the WHO ISARIC CCP-UK prospective cohort study, in which the authors assessed whether immunocompromised patients with COVID-19 were at a greater risk of in-hospital death and how this risk changed during the pandemic. The study included patients > 19 years of age with symptomatic community-acquired COVID-19. The researchers defined immunocompromised as immunosuppressant medication pre-admission, cancer treatment, organ transplant, HIV, or congenital immunodeficiency. The study found that immunocompromised patients were at an elevated risk of in-hospital mortality compared with immunocompetent patients, even after adjusting for age, sex, deprivation, ethnicity, vaccination, and comorbidities. However, not all immunocompromising conditions had the same risk: patients with cancer had a higher risk of death but were less likely to have their care escalated to intensive care or ventilation. As the pandemic progressed, in-hospital mortality reduced more slowly for immunocompromised patients than for immunocompetent patients, especially with increasing age. The authors suggested that targeted measures, such as additional vaccine doses, monoclonal antibodies, and non-pharmaceutical preventive interventions, should be continually encouraged for immunocompromised patients [18]. Findings from another recent study suggested that OTRs are at increased risk for COVID-19, with a reported in-hospital mortality rate of 20–30% [19]. That study reported on the initial experience of OTRs who contracted SARS-CoV-2 infection in two centers during the first 3 weeks of the outbreak in New York City. They found that OTRs with COVID-19 illness had more severe outcomes, with 18% overall mortality, 24% of hospitalized patients, and 52% of ICU patients. That study suggested that COVID-19 has the potential to severely impact OTRs. This high mortality rate may be explained by their impaired immune response, with standard transplant immunosuppression targeting the adaptive immune system by predominately inhibiting interleukin-2. This inhibition results in impaired T-cell function and lymphopenia. T-cell function is important for controlling viral replication, and previous research has suggested that lymphopenia, a well-known adverse effect of immunosuppression, is associated with worse COVID-19 outcomes [20]. Perhaps in contrast to this expectation, the majority of OTRs in the present study recovered from COVID-19, a few were discharged from the hospital, and only three died. This finding is consistent with a recent study reporting that although COVID-19 illness among OTRs is more severe than that of the general population, the majority of these patients recover after a prolonged clinical course and virus shedding [14]. In our cohort, three patients from the OTR group died; one of them was a hepatitis C survival and renal transplantation patient. This patient died of post-septic shock after being treated with heavy antimicrobial and anti-inflammatory drugs. Our findings indicate the management of acutely infected patients in the OTR group needs to be enhanced with shotgun sequencing to understand the patient’s current disease and its risk for therapy resistance.

In the present study, we compared the demographic and clinical characteristics of OTRs with patients without organ transplants with or without comorbid conditions to enhance the understanding of COVID-19 disease in OTRs. Our findings indicated that OTRs were mostly male, and patients without comorbidities were mostly female. Patients without comorbidities had the best outcome, whereas most deceased patients had comorbidity. Severe disease was detected only among patients with comorbidity or among OTRs. Hospitalization and ICU admission were more common among patients with comorbid conditions or among OTRs. Diabetes, hypertension, or immunocompromised were most common among OTRs. The Ct value for PCR was highest in patients with comorbid conditions and in patients without comorbidities. This finding may indicate that, in our study, underlying clinical conditions rather than the Ct value influenced the clinical outcome. Immunocompromised patients had an overall higher Ct value compared to other patient groups.

Vaccination status did not differ significantly between the three clinical groups. These findings provide valuable insights into the differences and similarities of COVID-19 illness among OTRs and patients with or without comorbid conditions. Our data are in accordance with several studies that reported that most OTRs are males, with a mean age of 53 years [21]. Age was also a significant factor in our cohort, with the oldest patients belonging to the group with comorbid conditions and the youngest patients belonging to the group without comorbid conditions. Patient outcomes significantly differed across the three patient clinical groups in our cohort: all patients without comorbid conditions recovered, whereas most deceased patients had comorbid conditions.

We found no significant differences for either vaccination type or dose among the three patient clinical groups. Several studies have investigated the neutralization of VOCs with regard to vaccination status. For example, Planas et al. isolated a Delta variant from a traveler returning from India [22]. They compared the sensitivity of the Delta variant and other viral strains to monoclonal antibodies and to antibodies present in sera from individuals who were convalescent from COVID-19 illness or who were vaccine recipients. The Delta variant was resistant to neutralization by some anti-N-terminal domain and anti-receptor binding domain (RBD) monoclonal antibodies, indicating immune evasion to antibodies targeting non-RBD and RBD spike epitopes. When evaluating the sera of individuals who had received a single dose of the Pfizer-BioNTech or Oxford-AstraZeneca vaccines, the team discovered that these vaccines only marginally inhibited the Delta variant [15]. Wall et al. evaluated vaccine-induced neutralizing antibody escape by the Delta variant and compared the activity to previous strains with population-based vaccine efficacy estimates. For recipients of a single dose, the neutralizing antibody titers were significantly lower against the Beta and Delta VOCs compared with Alpha VOCs, indicating that although a single dose may provide significantly more protection than no vaccination, single-dose recipients are likely to be less protected against these SARS-CoV-2 variants [23]. A study in Israel evaluated the spread of the Delta variant and other volatile organic compounds in Europe [17]. Microneutralization assays using sera from 36 healthcare workers (31 women) who received the Pfizer-BioNTech vaccine revealed a significant decrease in Beta, Gamma, and Delta neutralizing titers compared with the original virus. The decreased neutralizing titer for the Alpha variant was not statistically significant. The remaining neutralization capacity conferred by the Pfizer-BioNTech vaccine against the Delta variant and other VOCs was likely protective despite being lower [17,18,19,20,21]. The authors of a review have discussed how newly emerging variants of SARS-CoV-2 pose high global public health concerns and impact the COVID-19 pandemic. Some variants have the ability to spread quickly across many countries and cause severe disease, while others may lessen the efficacy of current COVID-19 vaccines and immunotherapies, leading to breakthrough infections [24]. The focus of that review is on the Omicron variant, including its lineages and hybrid variants, and the genetic changes and underlying molecular mechanisms behind its higher transmissibility and immune escape. The article discusses concerns regarding the efficacy of currently available immunotherapeutics and vaccines, transmissibility, disease severity, and mortality associated with the Omicron variant. The article also presented challenges and strategies to counter the Omicron variant, its lineages, and hybrid variants amid the ongoing COVID-19 pandemic [24].

The present study has some limitations, most notably the relatively small sample size of the cohort. While the study provides valuable insights into the impact of COVID-19 on OTRs, the small sample size means that the results may not be representative of the wider population. Therefore, further studies using whole-genome sequencing are needed to track the evolution of the virus and its potential impact on vulnerable populations. Such studies would provide a more comprehensive understanding of how the virus spreads and affects immunocompromised individuals, allowing for more effective prevention and treatment strategies.

## 5. Conclusions

The findings of this cohort study underline the importance of implementing an active surveillance system in a hospital of tertiary care to assist in providing optimal medical care to immunocompromised patients. The results showed that OTRs had a higher number of unusual SARS-CoV-2 mutations in mild disease than the other clinical groups without organ transplants, suggesting that the genetic makeup of the virus may be more diverse in OTRs. This increased diversity may be due to immunosuppression and prolonged exposure to the virus. This research highlights the urgent need for increased genomic and epidemiological surveillance systems in hospitals to protect immunocompromised patients.

## Figures and Tables

**Table 1:** Demographic and clinical characteristics of 87 patients at Jeddah King Faisal Specialist Hospital and Research Center from 1 April 2021 to 31 March 2022, by clinical group.

	Patient Clinical Group			
Characteristic	Without Comorbid Condition, No. (%) (*n* = 30)	With Comorbid Condition, No. (%) (*n* = 38)	OTR, No. (%) (*n* = 19)	F ANOVA or *χ*^2^, *p*-Value
Sex				
Female (*n* = 49)	23 (26.4)	19 (21.8)	7 (8.1)	0.0136 *
Male (*n* = 38)	7 (8.1)	19 (21.8)	12 (13.8)	
Age (mean, SD), years	34.2 (15.6)	52.5 (22.3)	47.1 (16.9)	0.0008 *
Nationality				
Saudi (*n* = 48)	15 (28.9)	19 (36.5)	14 (26.9)	0.065
Non-Saudi (*n* = 4)	0	4 (7.7)	0	
UNK = 35 ^a^				
Tobacco smoking status				
No (*n* = 79)	30 (34.5)	32 (36.8)	17 (19.5)	0.079
Yes (*n* = 8)	0	6 (6.9)	2 (2.3)	
Patient outcome				
Recovered (*n* = 61)	30 (34.5)	23 (26.4)	8 (9.2)	0.0003 *
Released (*n* = 15)	0	7 (8.1)	8 (9.2)	
Hospitalized (*n* = 1)	0	1 (1.2)	0	
Deceased (*n* = 10)	0	7 (8.1)	3 (3.5)	
Disease severity				
Mild (*n* = 62)	26 (33.3)	25 (32.1)	11 (14.1)	0.0037 *
Severe (*n* = 16)	0	9 (11.5)	7 (8.9)	
Hospital admission				
Outpatient (*n* = 60)	28 (36.4)	23 (29.9)	9 (11.7)	0.002 *
Hospitalized (*n* = 17)	0	12 (15.6)	5 (6.5)	
UNK = 10 ^a^				
Length of hospital stay				
Not needed (*n* = 60)	28 (32.2)	24 (26.4)	9 (10.3)	0.0057 *
Short, <20 days (*n* = 12)	2 (2.3)	6 (6.9)	4 (4.6)	
Long, >20 days (*n* = 15)	0	9 (10.3)	6 (6.9)	
Intensive care unit admittance				
Yes (*n* = 16)	0	9 (10.3)	7 (8.1)	0.0028 *
No (*n* = 71)	30 (34.5)	29 (33.3)	12 (13.8)	
Variant detected				
Non-VOC (*n* = 4)	2 (2.3)	1 (1.2)	1 (1.2)	0.26
Alpha (*n* = 2)	1 (1.2)	0	1 (1.2)	
Beta (*n* = 2)	0	1 (1.2)	1 (1.2)	
Kappa (*n* = 3)	0	2 (2.3)	1 (1.2)	
Delta (*n* = 16)	4 (4.6)	5 (5.8)	7 (8.1)	
Omicron (*n* = 60)	23 (26.4)	29 (33.3)	8 (9.2)	
Diabetes mellitus				
No (*n* = 63)	30 (34.5)	24 (27.6)	9 (10.3)	<0.0001 *
Yes (*n* = 24)	0	14 (16.1)	10 (11.5)	
Hypertension				
No (*n* = 55)	30 (34.5)	19 (21.8)	6 (6.9)	<0.0001 *
Yes (*n* = 32)	0	19 (21.8)	13 (14.9)	
Immunocompromised				
No (*n* = 53)	30 (34.9)	22 (25.6)	1 (1.2)	<0.0001 *
Yes (*n* = 33)	0	15 (17.4)	18 (20.9)	
Ct value				
High (*n* = 0)	0	0	0	0.0498 *
Low (*n* = 67)	23 (26.4)	33 (37.9)	11 (12.6)	
Moderate (*n* = 20)	7 (8.1)	5 (5.8)	8 (9.2)	
Vaccination status				
Vaccinated (*n* = 42)	16 (33.3)	17 (35.4)	9 (18.8)	0.85
Unvaccinated (*n* = 6)	3 (6.3)	2 (4.2)	1 (2.1)	
UNK = 39 ^a^				
Type of vaccine (*n* = 42)				
BNT162b2 (*n* = 21)	9 (25.0)	7 (19.4)	5 (13.9)	0.44
ChAdOx1 (*n* = 11)	4 (11.1)	6 (16.7)	1 (2.8)	
Mixture of vaccines and other types (*n* = 4)	3 (8.3)	1 (2.8)	0	
UNK = 6 ^a^				
Vaccine breakthrough disease				
After first dose (*n* = 7)	2 (5.0)	3 (7.50)	2 (5.0)	0.85
After second dose (*n* = 23)	9 (22.5)	10 (25.0)	4 (10.0)	
After booster (*n* = 10)	5 (12.50)	4 (10.0)	1 (2.50)	
UNK = 2 ^a^				
Wave				
Delta (*n* = 25)	6 (6.9)	8 (9.2)	11 (12.6)	0.0064 *
Omicron(*n* = 62)	24 (27.6)	30 (34.5)	8 (9.2)	

Abbreviations: ANOVA, analysis of variance; Ct, cycle threshold; OTR, organ transplant recipient. * Indicates statistical significance of *p* < 0.05. (^a^): Unknown data were not included in the analysis.

**Table 2:** Demographic and clinical characteristics of 92 patients by variant wave from 1 April 2021 to 31 March 2022 at Jeddah King Faisal Specialist Hospital and Research Center.

Characteristic	Delta Wave, No. (%) (*n* = 26)	Omicron Wave, No. (%) (*n* = 66)	*t*-Test or *χ*^2^, *p*-Value
Sex			
Female (*n* = 52)	17 (18.5)	35 (38.0)	0.28
Male (*n* = 40)	9 (9.8)	31 (33.7)	
Age (mean, SD), years	49.3 (19.3)	42.2 (17.6)	0.125
Nationality			
Saudi (*n* = 48)	17 (32.1)	32 (60.4)	0.54
Non-Saudi (*n* = 4)	2 (3.8)	2 (3.8)	
UNK = 40 ^a^			
Tobacco smoking status			
No (*n* = 79)	24 (27.6)	55 (63.2)	0.28
Yes (*n* = 8)	1 (1.2)	7 (8.1)	
UNK = 5 ^a^			
Patient outcome			
Recovered (*n* = 62)	18 (20.5)	44 (50)	0.65
Released (*n* = 15)	6 (6.8)	9 (10.2)	
Hospitalized (*n* = 1)	0	1 (1.1)	
Deceased (*n* = 10)	2 (2.3)	8 (9.1)	
UNK = 4 ^a^			
COVID-19 disease severity			
Mild (*n* = 62)	18 (23.1)	44 (56.4)	0.261
Severe (*n* = 16)	7 (9.0)	9 (11.5)	
UNK = 14 ^a^			
Length of hospital stay			
Not needed (*n* = 60)	16 (18.4)	44 (50.6)	0.82
Short, <20 days (*n* = 12)	4 (4.6)	8 (9.2)	
Long, >20 days (*n* = 15)	5 (5.8)	10 (11.5)	
UNK = 5 ^a^			
Intensive care unit admittance			
Yes (*n* = 16)	6 (6.9)	10 (11.5)	0.39
No (*n* = 71)	19 (21.8)	52 (59.8)	
UNK = 5 ^a^			
Patient group			
No comorbidity (*n* = 30)	6 (6.9)	24 (27.6)	0.0064 *
Comorbidity (*n* = 38)	8 (9.2)	30 (34.5)	
OTR (*n* = 19)	11 (12.6)	8 (9.2)	
UNK = 5 ^a^			
Comorbidity			
No (*n* = 30)	6 (6.9)	24 (57.6)	0.192
Yes (*n* = 57)	19 (21.8)	38 (43.7)	
UNK = 5 ^a^			
Diabetes mellitus			
No (*n* = 63)	14 (16.1)	49 (56.3)	0.029 *
Yes (*n* = 24)	11 (12.6)	13 (14.9)	
UNK = 5 ^a^			
Hypertension			
No (*n* = 55)	14 (16.1)	41 (47.1)	0.375
Yes (*n* = 32)	11 (12.6)	21 (24.1)	
UNK = 5 ^a^			
Immunocompromised			
No (*n* = 53)	12 (13.9)	41 (47.7)	0.096
Yes (*n* = 33)	13 (15.1)	20 (23.3)	
UNK = 6 ^a^			
Ct value			
High (*n* = 1)	0	1 (1.1)	0.682
Low (*n* = 67)	18 (20.0)	50 (55.6)	
Moderate (*n* = 20)	7 (7.8)	14 (15.6)	
UNK = 4 ^a^			
Vaccination Status			
Vaccinated (*n* = 42)	12 (25)	30 (62.5)	0.0087 *
Unvaccinated (*n* = 6)	5 (10.4)	1 (2.1)	
UNK = 44 ^a^			
Type of Vaccine (*n* = 42)			
BNT162b2 (*n* = 21)	6 (16.7)	15 (41.7)	0.812
ChAdOx1 (*n* = 11)	2 (5.6)	9 (25)	
Mixture of vaccines and other types (*n* = 4)	1 (2.8)	3 (8.3)	
UNK = 6 ^a^			
Vaccine breakthrough disease			
After first dose (*n* = 7)	5 (12.5)	2 (5)	0.0032 *
After second dose (*n* = 23)	5 (12.5)	18 (45)	
After booster (*n* = 10)	0	10 (25)	
UNK = 6 ^a^			

Abbreviations: Ct, cycle threshold; OTR, organ transplant recipient. * Indicates statistical significance at *p* < 0.05. (^a^): Unknown data were not included in the analysis.

**Table 3:** SARS-CoV-2 variants associated with antiviral treatment resistance detected in organ transplant recipients.

Sample ID	Spike mAb-RM	3CLpro Mutation	RdRP Mutation	No. of Mutations	No. of Unusual Mutations	No. of Spike Mutations	No. of Unusual Spike Mutations
**13-Beta-Aug-21-ICU-VAC-OTR**	K417N, E484K, and N501Y	K90R	A185V and P323L	28	0	11	0
**15-Kappa-Aug-21-R-OTR**	E484K	None	P323F and K780R	23	1	9	0
**16-Delta-Aug-21-R-VAC-OTR**	L452R	None	P323L and G671S	31	2	11	1
**18-Delta-Aug-21-ICU-VAC-OTR**	L452R	None	P323L and G671S	34	4	9	1
**19-Delta-Aug-21-ICU-VAC-OTR**	L452R	None	P323L and G671S	53	17	13	2
**23-Delta-Sep-21-R-VAC-OTR**	L452R	None	P323L and G671S	29	0	9	0
**25-Delta-Sep-21-ICU-VAC-OTR**	L452R	None	M196I, P323L, and G671S	29	0	11	0
**32-Omicron-Jan-22-R-OTR**	R346K, S371L, K417N, N440K, G446S, E484A, Q493R, G496S, N501Y, N856K, and N969K	P132H	P323L	71	21	53	21
**4-Alpha-April-21-R-Non-VAC-OTR**	N501Y	None	P323L	68	37	10	1
**40-Omicron-Jan-22-R-OTR**	R346K, S371L, K417N, N440K, G446S, N856K, and N969K	P132H	P323L	49	5	30	5
**56-Omicron-Jan-22-R-OTR**	R346K, S371L, K417N, N440K, G446S, N856K, and N969K	P132H	P323L	44	1	25	1
**8-Non-VOC-April-21-R-OTR**	N501T	P96L	P323L	16	1	4	0
**82-Omicron-Feb-22-R-VAC-OTR-adm**	S371F, D405N, K417N, N440K, and N969K	P132H, I200V	P323L	49	1	23	0
**83-Omicron-Feb-22-ICU-OTR-adm**	R346K, S371L, K417N, N440K, G446S, N856K, and N969K	P132H	P323L	44	0	24	0
**86-Omicron-March-22-ICU-VAC-OTR-adm**	R346K, S371L, K417N, N440K, G446S, E484A, Q493R, G496S, N501Y, N856K, and N969K	P132H	P323L	49	0	31	0
**88-Omicron-March-22-R-OTR-adm**	S371L, K417N, N440K, G446S, N856K, and N969K	P132H	D235E and P323L	70	25	48	22
**90-Omicron-Feb-22-ICU-OTR-adm**	R346K, S371L, K417N, N440K, G446S, N856K, and N969K	P132H	P323L	46	1	28	1

Abbreviation: mAb-RM spike monoclonal antibody resistance mutation. Detected variants were compared with data in the Stanford Coronavirus Antiviral and Resistance Database. Red shaded cells in the table indicate a deceased patient. Sample ID reflects patient data in the following order: sample identification number, variant type, month and year of infection, regular hospital (R) or intensive care unit (ICU) admission, vaccinated (VAC) or unvaccinated (UV), and organ transplant recipient (OTR).

**Table 4:** SARS-CoV-2 variants associated with antiviral treatment resistance detected in deceased patients.

Sample ID	Spike mAb-RMs	3CLpro Mutation	RdRP Mutation	No. of Mutations	No. of Unusual Mutations	No. of Spike Mutations	No. of Unusual Spike Mutations
**25-Delta-Sep-21-ICU-VAC-OTR**	L452R	None	M196I, P323L, and G671S	29	0	11	0
**5-Kappa-Jun-21-ICU-VAC**	E484K	None	P323F	21	1	8	0
**76-Omicron-Feb-22-ICU-adm**	R346K, S371L, K417N, N440K, G446S, E484A, N501Y, N856K, and N969K	P132H	P323L	52	3	32	2
**77-Omicron-Feb-22-ICU-adm**	R346K, S371L, K417T, N440K, G446S, N856K, and N969K	P132H	P323L	48	6	28	5
**80-Omicron-Feb-22-ICU-VAC-adm**	R346K, S371L, K417N, N440K, L455F, E484A, Q493R, G496S, N501Y, N856K, and N969K	P132H	P323L and M794I	58	5	33	1
**83-Omicron-Feb-22-ICU-OTP-adm**	R346K, S371L, K417N, N440K, G446S, N856K, and N969K	P132H	P323L	44	0	24	0
**84-Omicron-Feb-22-ICU-adm**	R346K, S371L, K417N, N440K, G446S, L452M, N856K, and N969K	P132H	P323L	44	0	25	0
**86-Omicron-March-22-ICU-VAC-OTP-adm**	R346K, S371L, K417N, N440K, G446S, E484A, Q493R, G496S, N501Y, N856K, and N969K	P132H	P323L	49	0	31	0
**87-Omicron-Feb-22-ICU-VAC-adm**	S371L, K417N, N440K, G446S, N856K, and N969K	P132H	P323L	64	23	46	23
**91-Omicron-March-22-R-adm**	S371F, D405N, K417N, N440K, E484A, Q493R, N501Y, and N969K	P132H	P323L	54	1	28	0

Abbreviation: mAb-RM spike monoclonal antibody resistance mutation. Detected variants were compared with data in the Stanford Coronavirus Antiviral and Resistance Database. Sample ID reflects patient data in the following order: sample identification number, variant type, month and year of infection, regular hospital (R) or intensive care unit (ICU) admission, vaccinated (VAC) or unvaccinated (UV), and organ transplant recipient (OTR).

## Data Availability

The data and codes used in this study are available on request. The SARS-CoV-2 whole genome sequencing data files were deposited on the GISAID website.
